# Professionelle Gesundheitskompetenz. Berufs- und geschlechtsspezifische Aspekte

**DOI:** 10.1007/s00103-025-04014-8

**Published:** 2025-02-18

**Authors:** Lennert Griese, Doris Schaeffer

**Affiliations:** 1 Fakultät für Gesundheitswissenschaften, Universität Bielefeld, Universitätsstraße 25, 33615 Bielefeld, Deutschland; 2 Hertie School, Berlin, Deutschland

**Keywords:** Gesundheitsinformation/-kommunikation, Qualifikation, Gesundheitsprofessionen/-berufe, Ärztinnen/Ärzte, Pflege, Health information/communication, Qualification, Health professionals/professions, Physicians, Nursing

## Abstract

**Hintergrund:**

Die Bedeutung der Gesundheitsprofessionen (GP) für die Förderung von Gesundheitskompetenz (GK) wird als zunehmend wichtig diskutiert. Ziel des Artikels ist es, die professionelle GK ausgewählter GP zu analysieren und zu fragen, wo die Schwierigkeiten bei der Förderung der GK bestehen, welche berufs- und geschlechtsspezifischen Unterschiede sichtbar werden und wie die Aufgabenrealisierung mit organisatorischen und qualifikatorischen Rahmenbedingungen zusammenhängt.

**Methode:**

Im Mai und Juni 2022 wurden 624 Pflegefachpersonen und 297 Ärzt:innen mit einem neu entwickelten Fragebogen, der die professionelle GK mit 34 Items in 4 Aufgabenbereichen misst (HLS-PROF‑Q), online befragt. Die Befragten wurden über zwei Online-Panels rekrutiert. Es wurden Scores professioneller GK (0–100 Punkte) sowie die subjektiven Schwierigkeiten bei der Aufgabenbewältigung berechnet. Die Analyse der Schwierigkeiten und Zusammenhänge erfolgte mittels deskriptiver und bivariater Statistik.

**Ergebnisse:**

Die GP erreichen je nach Aufgabenbereich durchschnittlich 54,0 bis 73,8 von 100 möglichen Punkten. Die Aufgaben in den Bereichen „professionelle digitale GK“ und „Informations- und Wissensvermittlung“ werden am schwierigsten eingeschätzt. Insgesamt zeigen sich kaum Unterschiede zwischen den Berufsgruppen. Vereinzelt werden Unterschiede nach Geschlecht sichtbar. Die Bewältigung der Aufgaben bei der Förderung der GK ist positiv mit den untersuchten organisatorischen und qualifikatorischen Rahmenbedingungen assoziiert.

**Diskussion:**

Die professionelle GK fällt moderat aus. Die Ergebnisse deuten zugleich auf Optimierungsbedarf und geben konkrete Hinweise, wie die professionelle GK gestärkt werden kann und welche Anpassungen der organisatorischen und qualifikatorischen Rahmenbedingungen erforderlich sind.

**Zusatzmaterial online:**

Zusätzliche Informationen sind in der Online-Version dieses Artikels (10.1007/s00103-025-04014-8) enthalten.

## Abstract (en)

**Background:**

The importance of health professionals (HPs) in promoting health literacy (HL) is being increasingly emphasized. This article aims to analyze the professional HL of selected HPs, examine professional and gender-specific differences, and identify the challenges in promoting HL as well as its relationship to organizational and qualification-related conditions.

**Method:**

In May and June 2022, 624 nursing professionals and 297 physicians were surveyed online using a newly developed questionnaire, which measures professional health literacy through 34 items across four key domains (HLS-PROF‑Q). Participants were recruited via two online panels. Scores for professional health literacy (ranging from 0–100) and the perceived difficulties in managing tasks were examined. The analysis of perceived difficulties in promoting HL and associations with organizational and qualification-related conditions was conducted using descriptive and bivariate statistics.

**Results:**

Depending on the domain, HPs achieved an average of 54.0 to 73.8 out of 100 possible points. The tasks in the domains “professional digital HL” and “conveying information and knowledge” were considered the most challenging. Overall differences between the two HP groups were minimal, though some gender-specific differences were observed. Managing tasks involved in promoting HL was positively associated with the organizational and qualification-related conditions examined.

**Discussion:**

The results show that professional HL is moderate. However, they also highlight areas for improvement and provide insights into where professional HL can be strengthened as well as which adjustments to organizational and qualification-related conditions are needed.

## Hintergrund

Mit dem Wandel zur Informations- und Wissensgesellschaft wächst die Herausforderung, sich in einer immer größeren Menge von Gesundheitsinformationen zurechtzufinden, in der zuverlässige Informationen und qualitativ fragwürdige Informationen nebeneinanderstehen [1–4]. Die Nutzung von Gesundheitsinformationen verlangt hohe Gesundheitskompetenz (GK) – verstanden als die Fähigkeit, die richtige Information, egal in welcher Form, zu finden, zu verstehen, ihre Vertrauenswürdigkeit einzuschätzen und sie für die Entscheidungsfindung oder das eigene Gesundheitsverhalten zu nutzen [5]. GK wird in allen Lebensbereichen benötigt – im Bildungs‑, Ernährungs‑, Konsum- und Freizeitbereich und ganz besonders, wenn es um konkrete Gesundheits- und Krankheitsfragen geht. Hier sind insbesondere die Gesundheitsprofessionen (GP) gefordert. Doch auch für sie geht der Informations- und Wissenszuwachs mit neuen Anforderungen einher. Zugleich wird die Förderung von GK für sie zu einer immer wichtigeren Aufgabe, da der Anstieg an Gesundheitsinformationen weite Teile der Bevölkerung vor Schwierigkeiten stellt [6–8].

Während über die GK der Bevölkerung mittlerweile valide Daten vorliegen, existieren über die GK der GP bislang so gut wie keine empirischen Erkenntnisse. Das gilt speziell dann, wenn das Interesse nicht auf ihre persönliche, sondern die professionelle GK zielt, also auf die Aufgaben abgehoben wird, die sie als GP bei der professionellen Förderung der GK ihrer Patient:innen bewältigen müssen.

Um die professionelle GK zu untersuchen, wurden von einem Dreiländerkonsortium, bestehend aus Forscher:innen aus Deutschland (Hertie School, Universität Bielefeld), Österreich (Gesundheit Österreich GmbH) und der Schweiz (Careum), ein neues Konzept und Messinstrument entwickelt und erstmals eingesetzt [6]. In dem vorliegenden Artikel werden ausgewählte, damit für Deutschland gewonnene Daten dargestellt und diskutiert. Ziel ist es, zu analysieren:

- welche Aufgaben die befragten GP (Pflegefachpersonen und Ärzt:innen) bei der Förderung von GK als besonders schwierig einschätzen,
- welche berufs- und geschlechtsspezifischen Unterschiede sich dabei identifizieren lassen und
- inwieweit ausgewählte organisatorische und qualifikatorische Rahmenbedingungen mit der Aufgabenrealisierung in Beziehung stehen.

Zunächst erfolgt eine konzeptionelle Verortung der Studie.

### Konzeptionelle Rahmenüberlegungen

Für die Bevölkerung stellen die GP – trotz der Zunahme der Bedeutung des Internets und digitaler Medien – nach wie vor wichtigste Informationsinstanz bei Gesundheits- und Krankheitsfragen dar [9, 10]. Auch bei der Informationssuche, bei unverstandenen, schwer oder nicht einschätzbaren Gesundheitsinformationen sind sie eine wichtige Anlaufstelle, von der Verständnis, Klarstellung, ausreichend Zeit für Rückfragen und kompetente Unterstützung bei der Verarbeitung von Informationen erwartet wird [11]. Denn oft rufen gefundene Informationen eher Verwirrung als Erkenntnisgewinn hervor. Mit der Digitalisierung hat sich das verstärkt: Durch sie hat sich zwar der Zugang zu Informationen vereinfacht, aber zugleich ist die Menge an Informationen stark gewachsen. Auch die Qualität ist fragwürdiger geworden, weil auch Fehl- und Falschinformationen deutlich zugenommen haben. Die daraus erwachsenen Irritationen (etwa bei der Beurteilung von Gesundheitsinformationen) werden ebenfalls oft an die GP herangetragen, sodass die Förderung der GK für sie zu einer bedeutenden Aufgabe geworden ist.

Doch ist unklar, ob die GP sich in der Lage sehen, diese Aufgabe auch angemessen zu erfüllen, zumal sie, je nach Setting, vielfach nicht mit den gegebenen Rahmenbedingungen in Übereinstimmung zu bringen ist. Seit vielen Jahren wird beklagt, dass die Zeit für Informationsvermittlung und Kommunikation zu knapp ist, sie nicht ausreichend refinanziert wird, geeignete räumliche Bedingungen fehlen [12–15] und ebenso, dass die Kommunikation herausfordernd und aufwendig geworden ist – nicht zuletzt, weil Patient:innen immer höhere Ansprüche und Erwartungen an die GP herantragen [16–18].

Verstärkt wird dies durch den Wandel der Patientenrolle, der die GP speziell im Bereich Informationsvermittlung (und Kommunikation) mit Anforderungen konfrontiert, auf die sie nicht ausreichend vorbereitet und eingestellt sind, wie die jahrelange Diskussion über Shared Decision Making (SDM) exemplarisch andeutet – ein Thema, das als Kernelement der veränderten Patientenrolle gilt, dessen Implementation aber sehr zäh verläuft [19, 20].

Ähnlich ist es mit Veränderungen, die die Digitalisierung mit sich bringt. Gerade sie stößt im Gesundheitssystem und bei vielen GP auf ein schleppendes Anpassungsverhalten [21–24]. Angesichts des Deutschland seit Langem attestierten digitalen Entwicklungsrückstands verwundert dies nicht, beschränkt aber ebenfalls die Möglichkeiten der Unterstützung von Patient:innen im Umgang mit den mittlerweile meist digitalen Gesundheitsinformationen.

Die angeführten Aspekte unterstreichen, wie wichtig es für eine gelingende Stärkung der GK der Bevölkerung ist, die professionelle GK genauer zu erforschen. Bislang wurde das Thema kaum untersucht, sodass zunächst zu klären ist, wie professionelle GK zu definieren und konzeptualisieren ist. Damit wurde im Rahmen des Dreiländerkonsortiums begonnen und folgende Definition erarbeitet:

„Professionelle Gesundheitskompetenz umfasst die Motivation, das Wissen und die Fähigkeiten, professionell relevantes Wissen und Informationen in unterschiedlicher – auch digitaler – Form finden, verstehen, beurteilen und nutzen zu können, um im Berufsalltag professionell auf dem State of the Art agieren zu können und gesundheits- und krankheitsrelevantes Wissen und ebensolche Informationen so aufbereiten, vermitteln und kommunizieren zu können, dass sie von Patientinnen und Patienten verstanden, (kritisch) beurteilt und zur Entscheidungsfindung über Gesundheitsfragen genutzt werden können.“ [25, S. 17]

Wichtig zu betonen ist, dass diese Definition als Teil einer ganzen „Familie“ von GK-Definitionen zu verstehen ist [26]. Sie nimmt explizit auf die in Europa weitverbreitete Definition von Sørensen et al. [5] Bezug, nach der der Umgang mit den 4 Schritten der Informationsverarbeitung (gesundheitsrelevante Informationen finden, verstehen, beurteilen/einschätzen und anwenden) den Kern von GK bilden. Hervorzuheben ist außerdem, dass die professionelle GK, ebenso wie die generelle GK, als *relational* [27] zu verstehen ist, d. h., sie ist sowohl das Ergebnis der persönlichen Fähigkeiten der GP als auch der gegebenen situativen und strukturellen Bedingungen.

Übereinstimmend mit dieser Definition lassen sich 4 Aufgabenbereiche identifizieren, die bei der professionellen Förderung von GK durch die GP zu bewältigen sind:

1. Eine wichtige Aufgabe im Rahmen professioneller GK stellt ein systematisches, umfassendes professionelles *Informations- und Wissensmanagement* dar. Denn auch die GP sind mit einem enormen, rasant voranschreitenden (globalen) Informations- und Wissenszuwachs konfrontiert. Daher sind sie ihrerseits gefordert, sich ständig mit neuen (Fach‑)Informationen auseinanderzusetzen, um auf dem aktuellen professionellen Informations- und Wissensstand zu bleiben und in ihrer täglichen Arbeit nach dem jeweiligen State of the Art und der vorliegenden Evidenz handeln zu können und ebenfalls, um Patient:innen auf dem neuesten Wissensstand informieren und beraten zu können.
2. Nicht weniger bedeutsam ist es für sie, sich mit Fragen *der Informations- und Wissensvermittlung *auseinanderzusetzen, um in der Lage zu sein, professionelles Wissen und Informationen so zu vermitteln und zu erklären, dass Patient:innen diese Informationen auch verstehen, einschätzen und so nutzen können, dass ihre Gesundheit und ihre GK davon profitieren.
3. Eng damit verbunden ist die *patientenzentrierte Kommunikation* – eine Aufgabe, die zunehmend an Stellenwert gewonnen hat, um der veränderten Patientenrolle entsprechen zu können: nicht mehr über Patient:innen, sondern mit ihnen zu entscheiden, gemeinsam Behandlungs- und Versorgungsziele und -strategien zu beraten und diese auszuhandeln, Informationsasymmetrien abzubauen und der Kommunikation sowie der Informations- und Wissensvermittlung mehr Raum zu geben.
4. Mit der *professionellen digitalen GK* ist die gesellschaftlich zunehmend bedeutende, aber im Alltag der GP keineswegs einfach leistbare Aufgabe angesprochen, Patient:innen bei den vielfältigen Herausforderungen im Umgang mit digitalen Informationsmedien und -informationen zu unterstützen und ihnen vor allem bei der Beurteilung krankheits- und gesundheitsbezogener Informationen beratend zur Seite zu stehen.

Diese 4 Aufgaben stehen im Mittelpunkt des neu erarbeiteten Erhebungsinstruments (PROF-HL-Q).

## Methoden

### Messung professioneller Gesundheitskompetenz

Der PROF-HL‑Q misst die selbsteingeschätzten Schwierigkeiten bei der Erfüllung von insgesamt 34 Aufgaben in den zuvor dargelegten 4 Bereichen professioneller GK:

- Informations- und Wissensmanagement (7 Items),
- Informations- und Wissensvermittlung (17 Items),
- patientenzentrierte Kommunikation (6 Items) und
- professionelle digitale GK (4 Items).

Es wird gefragt, „wie einfach oder schwierig“ die Aufgaben für die teilnehmenden GP jeweils sind. Zu beantworten sind die Items auf einer 5‑stufigen Antwortskala („sehr schwierig“, „eher schwierig“, „weder einfach noch schwierig“, „eher einfach“, „sehr einfach“).

Für die Auswertung wurden die Antworten für jeden Aufgabenbereich summiert und in einen gemeinsamen Score von 0 bis 100 Punkten skaliert, wobei 0 den geringsten und 100 den höchsten Wert darstellt. Ein höherer Score lässt somit auf geringere Schwierigkeiten bei der Aufgabenrealisierung schließen. Ein Score wurde nur dann berechnet, wenn alle Fragen der jeweiligen Itembatterie vollständig beantwortet wurden.

Das Erhebungsinstrument wurde im Rahmen der HLS-PROF-Studie auf seine psychometrischen Eigenschaften geprüft. Die Ergebnisse deuten auf zufriedenstellende Eigenschaften des Instruments [6]. (Mit einem Cronbachs Alpha von 0,82–0,90 für Pflegefachpersonen und 0,80–0,91 für Ärzt:innen weisen die Skalen zudem eine zufriedenstellende interne Konsistenz auf.)

In die Analyse gingen neben der professionellen GK 5 ausgewählte *organisatorische Rahmenbedingungen* ein. Dazu wurde gefragt, inwieweit

- ausreichend Zeit und
- geeignete Räumlichkeiten für Patientengespräche zur Verfügung stehen,
- die Möglichkeit besteht, ungestört Gespräche zu führen,
- zusätzliche Gespräche zur Klärung weiterführender Fragen angeboten werden können und
- auf Dolmetschdienste oder digitale Übersetzungsmöglichkeiten zurückzugriffen werden kann.

Außerdem wurde die Beurteilung der Ausbildung einbezogen: Dazu wurde gefragt, wie gut die Befragten durch ihre Ausbildung auf die bei der Förderung von GK anfallenden Aufgaben vorbereitet worden sind. Zusätzlich wurde danach gefragt, wie vertraut sie mit dem GK-Konzept sind. Eine Übersicht über die Verteilung der Antworten zu den Rahmenbedingungen, der Einschätzung der Ausbildung und der Vertrautheit mit dem GK-Konzept ist im Onlinematerial zu finden (Tab. Z1).

### Studiendesign und Studienpopulation

Bei der Untersuchung (HLS-PROF-GER) handelt es sich um eine quantitative Querschnittserhebung, in der Allgemeinärzt:innen bzw. hausärztlich tätige Internist:innen und Pflegefachpersonen der Gesundheits- und Krankenpflege mit regelmäßigem Patientenkontakt via computerassistierter Web-Interviews (CAWI) zu ihrer professionellen GK befragt wurden. Die Befragung wurde von Mai bis Juni 2022 durchgeführt. Für die Rekrutierung der Ärzt:innen und Pflegefachpersonen wurde jeweils auf ein Online-Panel zurückgegriffen. Über das „Sermo-Panel“, das auf den Austausch zwischen Mediziner:innen zielt, wurden 4468 Ärzt:innen per E‑Mail zur Befragung und zu einem initialen Screening (3 Fragen zur Sicherstellung des passenden Berufsabschlusses und des regelmäßigen Patientenkontakts) eingeladen. Von den 352 Personen, die sich am Screening beteiligten, erfüllten 326 die Einschlusskriterien und wurden zur Hauptbefragung weitergeleitet. Für die Ärzt:innen konnten 297 verwertbare Datensätze generiert werden. Die Rekrutierung der Pflegefachpersonen erfolgte in ähnlicher Weise über das „Payback Access-Panel“ und die Versendung des initialen Screening-Fragebogens. Von den 2644 eingeladenen Personen, wurden 1131 aus nicht bekannten Gründen nicht erreicht, 841 Personen erfüllten nicht die Einschlusskriterien. Letztendlich gingen 624 verwertbare Datensätze für die Zielgruppe der Pflegenden in die Analyse ein.

### Datenauswertung

Mit dem vorliegenden Artikel erfolgte eine Spezialauswertung des HLS-PROF-GER [28–30]. Dazu wurden in einem ersten Schritt die Scores professioneller GK berechnet und nach Berufsgruppen und dem Geschlecht stratifiziert. Unterschiede zwischen den Geschlechtern wurden mittels bivariater Analyse (t-Test, Mann-Whitney-U-Test) geprüft. In einem zweiten Schritt wurden die 8 von den Befragten als besonders schwierig eingeschätzten Aufgaben zur professionellen GK identifiziert. Dazu wurden die Antwortkategorien „eher schwierig“ und „sehr schwierig“ zusammengefasst und die Items nach Schwierigkeitsgrad geordnet. Potenzielle Unterschiede nach Geschlecht wurden mittels Z‑Tests untersucht. Für die Überprüfung des Zusammenhangs zwischen den 8 schwierigsten Aufgaben und den Rahmenbedingungen, der Bewertung der Ausbildung sowie der Vertrautheit mit dem GK-Konzept wurden Korrelationskoeffizienten nach Spearman berechnet. Alle Analysen wurden mit der Statistiksoftware SPSS Version 28 durchgeführt.

Für die Berechnungen wurde ein für das Geschlecht gewichteter Datensatz genutzt, um den vorhandenen Abweichungen zwischen der realisierten Stichprobe und der Grundgesamtheit der Ärzt:innen und Pflegefachpersonen besser zu entsprechen [31, 32]. Für die Berechnung der Korrelationskoeffizienten wurde der ungewichtete Datensatz genutzt.

## Ergebnisse

In der für das Geschlecht gewichteten Stichprobe sind insgesamt 930 Fälle (304 für Ärzt:innen und 626 für Pflegefachpersonen) enthalten. Dabei überwiegt der Anteil der Frauen deutlich gegenüber dem der Männer (Tab. 1). Im Mittel sind die Befragten 46 Jahre alt, wobei die befragten Ärzt:innen rund 11,5 Jahre älter sind als die befragten Pflegefachpersonen. Mit durchschnittlich 18,2 bzw. 19,6 Jahren weisen beide Berufsgruppen eine ähnlich lange Verweildauer im Beruf auf. Der Großteil der Befragten ist in Deutschland geboren. Gut zwei Drittel der Pflegenden sind in einer stationären Einrichtung tätig, rund 90 % der Ärzt:innen praktizieren in einem ambulanten Setting.

**Tab. 1:** Stichprobenbeschreibung

	Gesamt			Pflegefachpersonen			Ärzt:innen			
	*%*		*n*	*%*	*n*		*%*		*n*	
*Geschlecht*										
Weiblich	72,0	669		83,8		525	47,7			145
Männlich	26,9	251		15,8		99	50,0			152
Fehlende Werte	1,1	10		0,5		3	2,3			7
*Alter*										
*MW (SD), Min.-Max.*	*46,0 (12,9), 19–75*			*42,2 (12,6), 19–67*			*53,7 (10,1), 29–75*			
< 30 Jahre	14,3	133		21,2		133	0	0		
30–39 Jahre	19,1	177		23,0		144	10,9	33		
40–49 Jahre	21,1	197		22,8		143	17,8	54		
50–59 Jahre	26,7	248		20,6		129	39,2	119		
60 + Jahre	18,8	175		12,4		78	32,1	98		
*Jahre im Beruf*										
*MW (SD), Min.-Max.*	*18,6 (11,4), <* *1–46*			*18,2 (12,4), <* *1–46*			*19,6 (9,0), <* *1–45*			
0–5 Jahre	14,9	137		18,8		117	6,4	19		
6–10 Jahre	13,7	126		14,9		93	10,7	33		
11–20 Jahre	31,0	285		27,5		172	37,3	113		
21–30 Jahre	26,0	239		20,1		126	37,3	113		
30 + Jahre	14,4	133		17,2		107	8,3	25		
Fehlende Werte	1,7	10		1,7		10	–	–		
*Geburtsland*										
Deutschland	89,3	831		91,3		572	85,2	259		
Anderes	10,7	99		8,7		54	14,8	45		
*Setting*										
Niedergelassene Praxis, medizinisches Versorgungszentrum/Primärversorgungszentrum	40,9	380		17,4		109	89,4	271		
Krankenhaus/Rehabilitationsklinik	34,6	322		46,3		290	10,4	32		
Stationäre Pflegeeinrichtung	15,4	144		22,8		143	0,2	1		
Ambulante Pflege	7,2	67		10,8		67	0	0		
Anderes	1,8	17		2,7		17	0	0		

*Max.* Maximum, *Min.* Minimum, *MW* Mittelwert, *SD* Standardabweichung

Gewichteter Datensatz gesamt: *n* = 930, Pflegefachpersonen = 626; Ärzt:innen = 304

### Scores professioneller Gesundheitskompetenz

**Abb. 1:**
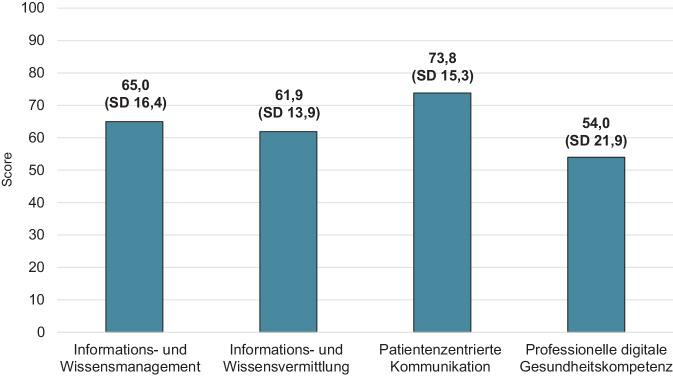
Scores in den 4 Aufgabenbereichen professioneller Gesundheitskompetenz für die Gesamtstichprobe, gewichtete Stichprobe (eigene Abbildung) SD, Standardabweichung, Informations- und Wissensmanagement: *n* = 903; Informations- und Wissensvermittlung: *n* = 847; patientenzentrierte Kommunikation: *n* = 897; professionelle digitale Gesundheitskompetenz: *n* = 889

Beide GP erreichen im Mittel zwischen 54,0 bis 73,8 Punkte in den 4 Aufgabenbereichen professioneller GK. Die niedrigsten Scores zeigen sich bei der professionellen digitalen GK, gefolgt von der Informations- und Wissensvermittlung mit durchschnittlich 61,9 Punkten. Etwas mehr Punkte werden im Bereich des Informations- und Wissensmanagements erzielt (65,0). Deutlich höher fällt die Punktzahl bei der patientenzentrierten Kommunikation aus (73,8), sie wird mit Abstand als am leichtesten eingeschätzt (Abb. 1).

**Abb. 2:**
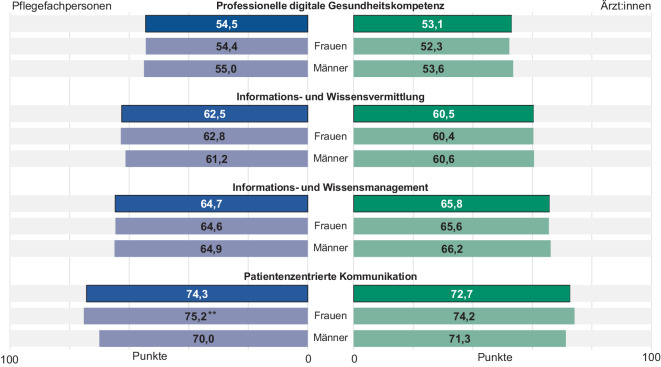
Die 4 Aufgabenbereiche professioneller Gesundheitskompetenz nach Berufsgruppen und Geschlecht, gewichtete Stichprobe (eigene Abbildung). *3* *Sternchen* *p* < 0,001, *2* *Sternchen* *p* < 0,01, *Sternchen p* < 0,05, Informations- und Wissensmanagement: Pflegefachpersonen *n* = 608, Ärzt:innen *n* = 295; Informations- und Wissensvermittlung: Pflegefachpersonen *n* = 559, Ärzt:innen *n* = 288; patientenzentrierte Kommunikation: Pflegefachpersonen *n* = 599, Ärzt:innen *n* = 298; professionelle digitale GK: Pflegefachpersonen *n* = 591, Ärzt:innen *n* = 298

Betrachtet man die beiden Berufsgruppen getrennt voneinander (Abb. 2), zeigt sich ein ähnliches Muster: Sowohl die Pflegefachpersonen als auch die Ärzt:innen erreichen bei der professionellen digitalen GK die geringsten (54,5 bzw. 53,1), bei der patientenzentrierten Kommunikation die höchsten Scores (74,3 bzw. 72,7). Die Informations- und Wissensvermittlung (62,5 bzw. 60,5) und Informations- und Wissensmanagement (64,7 bzw. 65,8) nehmen die Plätze 2 und 3 in der Rangfolge der schwierigsten Aufgabenbereiche ein.

Unter geschlechtsspezifischen Gesichtspunkten zeigen sich nur geringfügige Unterschiede. Einzig im Bereich der patientenzentrierten Kommunikation erreichen weibliche Pflegefachpersonen statistisch signifikant höhere Scores als männliche Befragte (MW: 75,2 vs. 70,0 Punkte, *p* < 0,01).

**Abb. 3:**
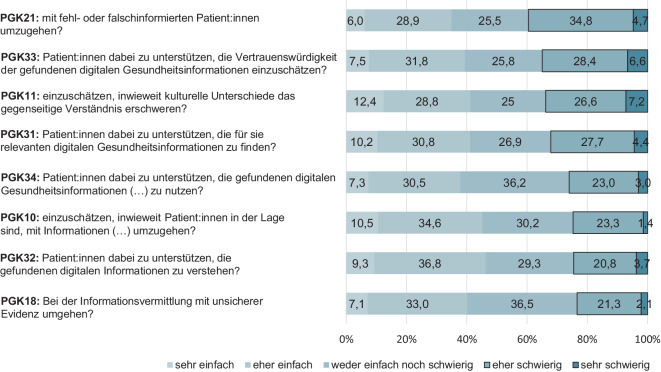
Die 8 Aufgaben, die im Rahmen der professionellen Gesundheitskompetenz (PGK) von den Befragten am häufigsten als „eher schwierig“ und „sehr schwierig“ bewertet wurden, gewichtete Stichprobe (eigene Abbildung)

Eine Betrachtung der 8 am häufigsten als „eher schwierig“ oder „sehr schwierig“ beurteilten Aufgaben (Abb. 3) zeigt für die Gesamtstichprobe, dass die Befragten am schwierigsten einschätzen, „mit fehl- oder falschinformierten Patient:innen umzugehen“ (PGK21: 39,5 %), gefolgt davon, Patient:innen bei der Beurteilung der Vertrauenswürdigkeit digitaler Informationen zu unterstützen (PGK33: 35,0 %) und „einzuschätzen, inwieweit kulturelle Unterschiede das gegenseitige Verständnis erschweren“ (PGK11: 33,8 %). An 4. und 5. Stelle stehen die Aufgaben, „Patient:innen dabei zu unterstützen, die für sie relevanten digitalen Gesundheitsinformationen zu finden“ (PGK31: 32,1 %) sowie diese „zur Verbesserung ihres Gesundheitsproblems oder ihrer Gesundheit zu nutzen“ (PGK34: 26 %). „Einzuschätzen, inwieweit Patient:innen in der Lage sind, mit Informationen über Krankheits- und Gesundheitsthemen umzugehen“ (PGK10), wird von 24,7 % der Befragten als eher/sehr schwierig eingeschätzt und steht damit an 6. Stelle. Das Item, „Patient:innen dabei zu unterstützen, die gefundenen digitalen Informationen zu verstehen“ (PGK32: 24,5 %), steht an vorletzter Stelle in der Liste der als am schwersten eingeschätzten Aufgaben, noch vor der Aufgabe „bei der Informationsvermittlung mit unsicherer Evidenz umgehen“ (PGK18: 23,4 %).

**Abb. 4:**
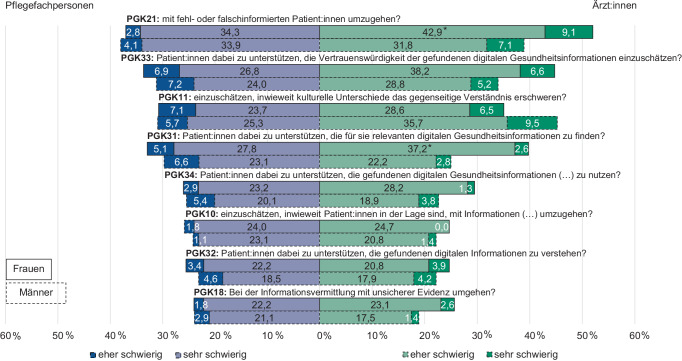
Anteile der Antworten („eher schwierig“ und „sehr schwierig“) für Pflegefachpersonen und Ärzt:innen nach Geschlecht zu den 8 schwierigsten Aufgaben im Rahmen der professionellen Gesundheitskompetenz (PGK), gewichtete Stichprobe (eigene Abbildung)

Bei der Untersuchung der geschlechtsspezifischen Unterschiede bei den 8 als am schwierigsten beurteilten Aufgaben (Abb. 4) wird deutlich, dass die Differenzen innerhalb der Gruppe der Pflegenden sehr gering sind. Bei den Ärzt:innen zeigen sich größere Unterschiede. Das Item PGK21, „mit fehl- oder falschinformierten Patient:innen umzugehen“, wird von den befragten Ärztinnen statistisch signifikant als schwieriger eingeschätzt als von den Ärzten (Kategorie „eher schwierig“: 42,9 % vs. 31,8 %, *p* < 0,049). Dies gilt auch für Item PGK31, „Patient:innen dabei zu unterstützen, die für sie relevanten digitalen Gesundheitsinformationen zu finden“ (Kategorie „eher schwierig“: 37,2 % vs. 22,2 %, *p* = 0,005). Auch die übrigen Aufgaben werden, wenngleich nicht signifikant, von weiblichen Befragten für schwieriger gehalten. Einzig „einzuschätzen, inwieweit kulturelle Unterschiede das gegenseitige Verständnis erschweren“ (PGK11), wird von Ärzten in der Tendenz als schwieriger beurteilt (signifikanter Unterschied in der Kategorie „eher einfach“: Ärztinnen: 37,7 % vs. Ärzte: 24,8 %, *p* = 0,017).

### Zusammenhangsanalyse

Die Korrelationskoeffizienten für den Zusammenhang zwischen der Vertrautheit mit dem GK-Konzept, der Beurteilung der Ausbildung und den Rahmenbedingungen mit den subjektiven Schwierigkeiten bei der Bewältigung der 8 schwierigsten Aufgaben im Rahmen der professionellen GK sind in Tab. 2 dargestellt. Insgesamt deutet die Analyse auf eine schwache bis moderate Korrelationen hin (min. ρ = 0,11, max. ρ = 0,32).

**Tab. 2:** Spearman-Korrelation zwischen den 8 schwierigsten Aufgaben im Rahmen der professionellen Gesundheitskompetenz (PGK) und der Kenntnis des Gesundheitskompetenzkonzepts, der Ausbildungsbewertung und den Rahmenbedingungen nach Berufsgruppen

	Pflegefachpersonen							Ärzt:innen						
	Konzept	Ausbildung	R1	R2	R3	R4	R5	Konzept	Ausbildung	R1	R2	R3	R4	R5
PGK21	0,15^*^	0,22^*^	0,16^*^	0,12^*^	0,11	0,21^*^	0,03	0,12	0,26^*^	0,15^*^	−0,01	0,15	0,14	0,15^*^
PGK33	0,25^*^	0,21^*^	0,15^*^	0,07	0,07	0,22^*^	0,10	0,25^*^	0,25^*^	0,10	−0,05	0,03	0,06	0,31^*^
PGK11	0,09	0,11^*^	0,16^*^	0,18^*^	0,11^*^	0,17^*^	0,07	0,20^*^	0,19^*^	0,20^*^	−0,03	0,17^*^	0,12	0,21^*^
PGK31	0,31^*^	0,19^*^	0,14^*^	0,14^*^	0,12^*^	0,21^*^	0,13^*^	0,19^*^	0,32^*^	0,04	−0,11	0,01	0,09	0,19^*^
PGK34	0,24^*^	0,25^*^	0,17^*^	0,14^*^	0,13^*^	0,22^*^	0,14^*^	0,26^*^	0,27^*^	0,10	0,03	0,07	0,14	0,30^*^
PGK10	0,14^*^	0,09	0,14^*^	0,09	0,06	0,10	0,12	0,18^*^	0,25^*^	0,28^*^	0,02	0,26^*^	0,16^*^	0,25^*^
PGK32	0,25^*^	0,19^*^	0,16^*^	0,14^*^	0,11^*^	0,22^*^	0,17^*^	0,22^*^	0,24^*^	0,01	−0,05	−0,00	0,02	0,21^*^
PGK18	0,19^*^	0,20^*^	0,17^*^	0,15^*^	0,16^*^	0,21^*^	0,12^*^	0,23^*^	0,17^*^	0,23^*^	0,10	0,26^*^	0,30^*^	0,22^*^

Vertrautheit mit dem GK-Konzept: „Wie vertraut sind Sie mit dem Konzept Gesundheitskompetenz?“ (1 = wenig oder nicht vertraut, 2 = vertraut oder sehr vertraut)

Bewertung der Ausbildung: „Wie gut hat Ihre Ausbildung Sie auf die Vermittlung und Erklärung von Informationen vorbereitet?“ (1 = sehr schlecht, 2 = eher schlecht, 3 = weder gut noch schlecht, 3 = eher gut, 4 = sehr gut)

Rahmenbedingungen: R1: „Haben Sie ausreichend Zeit für Gespräche mit Patient:innen?“ R2: „Stehen Ihnen geeignete Räumlichkeiten für Patient:innengespräche zur Verfügung?“ R3: „Ist es Ihnen möglich, Gespräche mit Patient:innen zu führen, ohne dabei gestört oder unterbrochen zu werden?“ R4: „Können Sie bei Bedarf zusätzliche Gespräche zur Klärung weiterführender Fragen anbieten?“ R5: „Haben Sie die Möglichkeit, bei Bedarf auf Dolmetschdienste oder auf digitale Übersetzungsmöglichkeiten (z. B. Dolmetsch-Apps) zurückzugreifen?“ (1 = nie, 2 = selten, 3 = manchmal, 4 = häufig, 5 = (fast) immer)

Ungewichteter Datensatz: Pflegefachpersonen: *n* = 586–623; Ärzt:innen: *n* = 291–297

Die *p*-Werte wurden mittels Bonferroni-Korrektur angepasst. Ein adjustierter *p*-Wert von < 0,007 gilt als signifikant (mit Stern gekennzeichnet)

Befragte, die angeben, vertraut oder sehr vertraut mit dem Konzept zu sein, schätzen fast alle Aufgaben als leichter ein. Dies gilt auch für die Einschätzung der Ausbildung: Eine als besser beurteilte Ausbildung geht in beiden Gruppen durchgängig mit geringeren Schwierigkeiten bei der Aufgabenbewältigung einher.

Pflegende, die über bessere zeitliche (R1) und räumliche (R2) Ressourcen verfügen und häufiger die Möglichkeit haben, zusätzliche Gespräche anzubieten (R4), schätzen die Aufgabenbewältigung fast immer als einfacher ein. Dies zeigt sich partiell auch bei den Ärzt:innen. Bei ihnen steht zudem die Möglichkeit, auf Dolmetschdienste oder digitale Übersetzungsoptionen zurückzugreifen (R5), durchgängig mit einer einfacheren Aufgabenbewältigung im Zusammenhang.

## Diskussion

Die Analyse zielte darauf, die professionelle GK zu untersuchen und zu fragen, wie die GP – konkret Pflegefachpersonen und Ärzt:innen – die Aufgaben einschätzen, die sich bei der Förderung von GK von Patient:innen in 4 zuvor konzeptualisierten Bereichen stellen. Dabei galt das Interesse den insgesamt am schwierigsten beurteilten Aufgaben und der Frage, welche berufs- und geschlechtsspezifischen Unterschiede sich dabei zeigen und ebenso, welche Bedeutung qualifikatorischen und organisatorischen Rahmenbedingungen bei der Aufgabenbewältigung zukommt.

Gezeigt wurde, dass die Berufsgruppen in den 4 erfragten Aufgabenbereichen mit 54 bis 74 von 100 möglichen Punkten verhältnismäßig hohe Scores erreichen und ihre professionelle GK recht positiv einzuschätzen ist – positiver als nach der vorliegenden Literatur zu erwarten war (z. B. [33–35]). Bei der Einordnung ist zu beachten, dass es sich bei der vorliegenden Untersuchung um eine Online-Befragung handelte, in der vermutlich einzelne Teilgruppen, etwa weniger Interessierte oder weniger onlineaffine Personen, nicht teilgenommen haben, was die Ergebnisse positiv beeinflusst haben dürfte. Gleichzeitig deuten die Scores auf Optimierungspotenzial hin, da ein nicht unerheblicher Anteil an Punkten nicht erreicht wurde.

Dies spiegelt sich auch in den untersuchten Schwierigkeiten wider: Auffällig ist, dass die Aufgaben, die beiden Berufsgruppen subjektiv die größten Schwierigkeiten bereiten, samt und sonders aus 2 Bereichen stammen, der „Informations- und Wissensvermittlung“ und der „professionellen digitalen GK“. Die in den beiden anderen Bereichen „Informations- und Wissensmanagement“ und „patientenzentrierte Kommunikation“ enthaltenen Aufgaben werden dementsprechend als leichter eingeschätzt. Dies könnte darauf zurückzuführen sein, dass die sich hier stellenden Aufgaben überwiegend als Routineaufgaben angesehen werden, die ohnehin im Alltag anfallen und bewältigt werden müssen. Zudem haben beide Aufgabenbereiche – insbesondere die Kommunikation – in den letzten Jahren verstärkte Aufmerksamkeit erfahren, um die seit vielen Jahren geäußerte Kritik aufzugreifen und Verbesserungen einzuleiten [36–38]. Dagegen stellen die „Informations- und Wissensvermittlung“ und „professionelle digitale GK“ – verstanden als Förderung von Patient:innen im Umgang mit digitalen Informationen – weniger beachtete bzw. neuere Aufgabenbereiche dar, die noch stärker in die Ausbildungen der GP einbezogen werden sollten. Hinzu kommt, dass sie nicht unmittelbar mit der Bewältigung von Krankheit und Gesundheits- und Funktionsbeeinträchtigungen in Verbindung stehen und daher vermutlich schwerer in Einklang mit dem Selbst- und Aufgabenverständnis und der professionellen Identität zu bringen sind. Gleichwohl stellen sie drängende Aufgaben im Alltag der GP dar, wie u.a. qualitative Studien zeigen [39–41].

Betrachtet man die 8 schwierigsten Aufgaben unter inhaltlichen Gesichtspunkten, wird deutlich, dass die zum Bereich „Informations- und Wissensvermittlung“ gehörenden Items sich entweder auf das Thema Herausforderungen bei der Informationsvermittlung beziehen oder sich mit der Ermittlung von Informationsvoraussetzungen befassen. Beides sind wichtige und anspruchsvolle Aufgaben im Rahmen systematischer Informationsvermittlungsprozesse, die in der Aus‑, Fort- und Weiterbildung der GP bislang eine bestenfalls periphere Rolle spielen und im Praxisalltag daher eher intuitiv und auf der Basis von persönlichem Erfahrungswissen wahrgenommen werden müssen. Dass hier Veränderungen erforderlich sind, wird durch die von beiden befragten Berufsgruppen als am schwierigsten beurteilte Aufgabe des Fragebogens unterstrichen: Fast 40 % betrachten es als (sehr) schwierig, mit fehl- oder falschinformierten Patient:innen umzugehen – eine Herausforderung, die in den letzten Jahren mit der um sich greifenden „Infodemie“ [4] an Bedeutung gewonnen hat. Hier ist eine komplexe Aufgabe zu bewältigen, die nicht allein mit schlichten, faktenorientierten Richtigstellungen, etwa bei Falschinformationen, gelöst werden kann, sondern meist auch eine Intervention in grundlegende Annahmen und Vorstellungen im Zusammenhang mit Krankheiten [42] verlangt, ebenso die Einleitung von Verlern- und Umlernprozessen bei Patient:innen. Das wiederum setzt die Fähigkeiten dazu voraus, Lernprozesse stimulieren und begleiten zu können, und erfordert spezielle edukative Kompetenzen [43], doch auch mehr Zeit als in alltäglichen Konsultationen hierzulande vorgesehen ist [12]. Die Dringlichkeit, hier Veränderungen herbeizuführen, wird dadurch unterstrichen, dass diese Aufgabe in allen 3 Ländern, in denen die professionelle GK bislang erhoben wurde, auf ähnlich große Schwierigkeiten stößt [6].

Die drittschwierigste Aufgabe (PGK11: „einzuschätzen, inwieweit kulturelle Unterschiede das gegenseitige Verständnis erschweren“) weist auf eine anders gelagerte Herausforderung hin. Mittlerweile verfügt gut jede vierte Person (28,7 %) in Deutschland über einen Migrationshintergrund, von denen rund 16 Mio. auf eine *eigene* Migrationserfahrung schauen [44]. Menschen mit Migrationshintergrund stellen eine sehr heterogene Gruppe dar. Sie unterscheiden sich u. a. hinsichtlich ihrer Gesundheits- und Krankheitserfahrungen und -vorstellungen. Das Gesundheitssystem stärker an diese Entwicklung anzupassen und es diversitäts- und kultursensibler zu gestalten, gehört zu den seit Längerem bestehenden Forderungen [45–47], deren Relevanz durch die Ergebnisse der vorliegenden Analyse erneut gestützt wird. Auch das verlangt Veränderungen auf der Kompetenzebene, ebenso allerdings – wie auch die Korrelationsanalyse andeutet – Veränderungen auf struktureller Ebene (etwa den Ausbau von Dolmetsch- und Sprachmittlungsdiensten oder der technischen Ausstattung, die eine Vereinfachung der sprachlichen Verständigung ermöglicht [6, 48]).

Die sprachliche Verständigung zu erleichtern, dürfte künftig durch die fortschreitende Digitalisierung und die Fortschritte im Bereich künstlicher Intelligenz (KI) strukturell möglich werden – auch im Gesundheitswesen. Das setzt jedoch voraus, dass die Anpassung an die digitale Transformation und auch an die damit einhergehenden neuen Aufgaben dort rascher voranschreitet als bislang. Denn gerade dem deutschen Gesundheitssystem wird hier nach wie vor eine zu geringe Flexibilität und ein großer Entwicklungsrückstand attestiert [21, 24].

Wie wichtig es ist, diesen Rückstand aufzuholen, deutet die vorliegende Untersuchung erneut an. Denn alle gestellten Fragen zum Bereich „professionelle digitale GK“ – dem am schwierigsten eingeschätzten Aufgabenbereich – sind in der Rangliste der schwierigsten Aufgaben zu finden. Besonders schwer ist es für die befragten Berufsgruppen, Patient:innen zu unterstützen, die Vertrauenswürdigkeit digitaler Informationen einzuschätzen oder für sie relevante Informationen überhaupt ausfindig zu machen. Dieses Ergebnis erstaunt angesichts der verwirrenden Informationsvielfalt nicht, könnte sich jedoch ebenfalls durch weitere Fortschritte im Bereich KI verändern – positiv wie auch negativ [49]. Ebenso gibt es Bemühungen, zu gemeinsamen Qualitätsstandards im Bereich digitaler Information zu gelangen [50]. Konkret absehbar sind diese Entwicklungen bislang nicht. So oder so bleibt daher als Aufgabe, die professionelle digitale GK zu stärken – nicht zuletzt, um die GK von Patient:innen besser fördern und die Krankheitsbewältigung stärker unterstützen und erleichtern zu können.

Berufs- und geschlechtsspezifische Unterschiede im Antwortverhalten sind insgesamt gering. Auch in Studien zur GK in der Allgemeinbevölkerung lassen sich, wenn überhaupt, nur geringe geschlechtsspezifische Unterschiede identifizieren [8, 26]. Die durchgeführte Analyse deutet dies ebenfalls für die professionelle GK an.

Ein genauerer Blick auf die Einzelitems zeigt dennoch einige Differenzen, insbesondere in der Berufsgruppe der Ärzt:innen. Bei ihnen zeichnen sich die meisten Abweichungen im Antwortverhalten ab. Am größten und statistisch signifikant sind sie bei dem Item PGK31: „Patienten dabei zu unterstützen, die für sie relevanten digitalen Informationen zu finden“. Doch auch PGK21: „mit fehl- oder falschinformierten Patient:innen umzugehen“, fällt Ärztinnen deutlich schwerer als ihren männlichen Kollegen. Dies deutet an, dass die Geschlechterdifferenzen bei den Ärzt:innen durchaus beachtet werden sollten. Zugleich fällt auf, dass Ärztinnen tendenziell häufiger als ihre männlichen Kollegen Schwierigkeiten bei der Unterstützung von Patient:innen im Umgang mit *digitalen* Informationen angeben. Dies passt zu anderen Studienergebnissen, nach denen weibliche Befragte ihre digitalen Fähigkeiten generell als geringer einschätzen als männliche Befragte [51]. Die Autor:innen führen dies auf eine höhere Technikaffinität bei Männern und bestehende Stereotype und Rollenmuster zurück, die sich möglicherweise auch im Antwortverhalten bei den Items zur professionellen digitalen GK widerspiegeln. Diese abzubauen und ein gleichwertiges digitales Empowerment für beide Geschlechter zu ermöglichen, scheint auch der professionellen digitalen GK zuträglich zu sein.

Insgesamt zeigt die Analyse, dass es aufschlussreich ist, die subjektiven Schwierigkeiten beider Berufsgruppen bei der Förderung der GK ihrer Patient:innen genauer in den Blick zu nehmen und ihnen bei der Interventionsentwicklung Aufmerksamkeit zu schenken.

Dies gilt auch für die festgestellten Zusammenhänge. Wenngleich sie eher schwach ausgeprägt sind und der weiteren Überprüfung bedürfen, deuten sie darauf, dass die professionelle GK mit den gegebenen Ausbildungs- und Rahmenbedingungen wie auch dem Ausmaß der Konzeptkenntnis in Beziehung steht – ein Ergebnis, das sich ähnlich in den anderen deutschsprachigen Ländern zeigt [6]. Dies mag erwartbar erscheinen, unterstreicht aber die Wichtigkeit, die Kenntnis des GK-Konzepts bei den GP zu verbessern und die Qualifikations- und Rahmenbedingungen unter der Frage zu prüfen, ob sie geeignet sind, die GP in die Lage zu versetzen, mit neuen, im Zuge des gesellschaftlichen Wandels an Relevanz gewinnenden Aufgaben umzugehen.

### Stärken und Limitationen

Mit der Analyse der professionellen GK und der hier vorgenommenen Teilanalyse erfolgte ein erster Schritt zur Untersuchung der bei der professionellen Förderung von GK bestehenden Schwierigkeiten und Optimierungspotenziale. Gleichzeitig wurde Weiterentwicklungsbedarf deutlich: So ist limitierend anzuführen, dass es sich bei der Befragung um eine Online-Erhebung handelte, die vermutlich mit Selektionseffekten einhergegangen ist und eine Repräsentativität der Stichprobe ausschließt. Zudem wurde die professionelle GK durch Selbsteinschätzungen erfasst, die nicht zwangsläufig die tatsächlichen Fähigkeiten widerspiegeln. Dies könnte auch Einfluss auf die untersuchten Zusammenhänge gehabt haben. Außerdem handelt es sich um eine Querschnittsbefragung, die keine Rückschlüsse auf kausale Zusammenhänge erlaubt.

## Fazit

Eine hohe professionelle GK wird zunehmend als wichtig für die Förderung der GK der Bevölkerung und auch die Umsetzung von gesundheitskompetenten Organisationen diskutiert. Zwar fällt die professionelle GK recht positiv aus, doch machen die dargestellten Ergebnisse auch deutlich, dass Maßnahmen zur Verbesserung erforderlich sind. Auch geben sie Hinweise, wo dabei jeweils anzusetzen ist. Sie belegen zugleich, dass dem relationalen Verständnis von GK folgend ein doppelgleisiges Vorgehen bei der Entwicklung von Verbesserungsmaßnahmen sinnvoll ist, das sowohl auf die Stärkung professioneller Fähigkeiten wie auch auf strukturelle Veränderungen zielt.

## Supplementary Information

Tab. Z1 Bewertung der Ausbildung, Vertrautheit mit dem Gesundheitskompetenzkonzept und organisatorische Rahmenbedingungen insgesamt und nach Berufsgruppen
