# Supplementary material for: Professional health literacy. Professional and gender-specific aspects
Source: Bundesgesundheitsblatt Gesundheitsforschung Gesundheitsschutz. 2025 Feb 18;68(3):282–92. [Article in German] doi: 10.1007/s00103-025-04014-8 (PMC11868220; doi:10.1007/s00103-025-04014-8)
Supplement: Supplementary file 1 — Tab. Z1 Bewertung der Ausbildung, Vertrautheit mit dem Gesundheitskompetenzkonzept und organisatorische Rahmenbedingungen insgesamt und nach Berufsgruppen [file 103_2025_4014_MOESM1_ESM.pdf]

Onlinematerial zum Artikel von Griese und Schaeffer im Bundesgesundheitsblatt Ausgabe 3/2025

Tab. Z1 Bewertung der Ausbildung, Vertrautheit mit dem Gesundheitskompetenz-Konzept und organisatorische Rahmenbedingungen insgesamt und nach Berufsgruppen

	Gesamt		Pflegefachpersonen		Ärzt:innen	
	%	n	%	n	%	n
**Ausbildungsbewertung**						
„Wie gut hat Ihre Ausbildung Sie auf die Vermittlung und Erklärung von Informationen vorbereitet?“						
sehr gut/eher gut	46,2	427	51,4	318	35,7	108
weder gut noch schlecht	31,8	295	32,5	202	30,5	93
eher schlecht/sehr schlecht	22,0	203	16,1	100	33,9	103
**Vertrautheit mit dem Konzept**						
„Wie vertraut sind Sie mit dem Konzept Gesundheitskompetenz“						
sehr vertraut	3,9	36	4,2	26	3,4	10
vertraut	33,0	306	33,9	212	31,0	94
wenig vertraut	46,9	435	46,1	289	48,4	146
nicht vertraut	16,2	150	15,8	99	17,2	52
**Organisatorische Rahmenbedingungen**						
R1 „Haben Sie ausreichend Zeit für Gespräche mit Patient:innen?						
(fast) immer/häufig	38,2	355	28,8	181	57,5	174
manchmal	35,3	328	37,0	232	31,8	96
selten/nie	26,5	246	34,1	213	10,8	32
R2 „Stehen Ihnen geeignete Räumlichkeiten für Patientengespräche zur Verfügung?“						
(fast) immer/häufig	56,9	524	39,8	246	92,0	278
manchmal	18,5	171	25,9	160	3,6	11
selten/nie	24,5	226	34,4	212	4,4	13
R3 „Ist es Ihnen möglich, Gespräche mit Patient:innen zu führen, ohne dabei gestört oder unterbrochen zu werden?						
(fast) immer/häufig	49,3	453	33,8	209	81,1	244
manchmal	23,0	211	28,9	179	10,8	33
selten/nie	27,7	255	37,3	230	8,1	24
R4 „Können Sie bei Bedarf zusätzliche Gespräche zur Klärung weiterführender Fragen anbieten?“						
(fast) immer/häufig	48,4	442	39,6	242	65,9	201
manchmal	34,7	317	38,9	237	26,2	80
selten/nie	17,0	155	21,5	131	7,9	24
R5 „Haben Sie die Möglichkeit, bei Bedarf auf Dolmetschdienste oder auf digitale Übersetzungsmöglichkeiten (z.B. Dolmetsch-Apps) zurückzugreifen?“						
(fast) immer/häufig	27,0	249	25,1	154	31,1	94
manchmal	26,4	243	24,7	152	29,9	91
selten/nie	46,6	429	50,3	310	39,0	119

Gewichteter Datensatz gesamt: n=930, Ärzt:innen=304; Pflegefachkräfte=626. Angegeben sind die gültigen Prozentwerte.
